# COVID-19: Heterogeneous Excess Mortality and “Burden of Disease” in Germany and Italy and Their States and Regions, January–June 2020

**DOI:** 10.3389/fpubh.2021.663259

**Published:** 2021-05-07

**Authors:** Peter Morfeld, Barbara Timmermann, J. Valérie Groß, Philip Lewis, Pierluigi Cocco, Thomas C. Erren

**Affiliations:** ^1^Institute and Policlinic for Occupational Medicine, Environmental Medicine and Prevention Research, University of Cologne, Cologne, Germany; ^2^Department of Medical Sciences and Public Health, Occupational Medicine Unit, University of Cagliari, Cagliari, Italy

**Keywords:** SARS-CoV-2/COVID-19, SMR, Germany, Italy, mortality excess, epidemiology

## Abstract

Total mortality and “burden of disease” in Germany and Italy and their states and regions were explored during the first COVID-19 wave by using publicly available data for 16 German states and 20 Italian regions from January 2016 to June 2020. Based on expectations from 2016 to 2019, simplified Standardized Mortality Ratios (SMRs) for deaths occurring in the first half of 2020 and the effect of changed excess mortality in terms of “burden of disease” were assessed. Moreover, whether two German states and 19 Italian cities appropriately represent the countries within the European monitoring of excess mortality for public health action (EuroMOMO) network was explored. Significantly elevated SMRs were observed (Germany: week 14–18, Italy: week 11–18) with SMR peaks in week 15 in Germany (1.15, 95%-CI: 1.09–1.21) and in week 13 in Italy (1.79, 95%-CI: 1.75–1.83). Overall, SMRs were 1.00 (95%-CI: 0.97–1.04) in Germany and 1.06 (95%-CI: 1.03–1.10) in Italy. Significant SMR heterogeneity was found within both countries. Age and sex were strong modifiers. Loss of life expectancy was 0.34 days (1.66 days in men) for Germany and 5.3 days (6.3 days in men) for Italy [with upper limits of 3 and 6 weeks among elderly populations (≥65 years) after maximum potential bias adjustments]. Restricted data used within EuroMOMO neither represents mortality in the countries as a whole nor in their states and regions adequately. Mortality analyses with high spatial and temporal resolution are needed to monitor the COVID-19 pandemic's course.

## Introduction

On December 31, 2019, the World Health Organization (WHO) received information of a cluster of viral pneumonia in Wuhan, China (1). One week later, severe acute respiratory syndrome coronavirus 2 (SARS-CoV-2) was identified (2, 3), which has been spreading worldwide since. On March 11, the WHO declared corona virus disease a global pandemic (2). Although numerous questions concerning short- and long-term health consequences of infection remain (4–7), it is clear that COVID-19 can result in death.

Both Germany and Italy have been significantly exposed to SARS-CoV-2, but Italy has been hit much harder (8). Analyses of nine regions (9, 10) evinced significant excess mortality in February and March not only in Lombardy, but also in other regions that were not considered as infection hotspots. In Germany, available mortality information suggested a much lower overall mortality during the first COVID-19-epidemic wave, from March to June 2020 (11, 12). Males were more affected than females, and older people more than younger (10, 13).

For public health, it is crucial to monitor and compare deaths attributable to the virus at national and international levels (14), but also at regional levels. In China, such higher spatial resolution allowed insights into how surveillance and emergency response capabilities varied across the country (15). In the US, examining mortality in 477 cities and 3,113 counties demonstrated risk variations that can inform the allocation of counter-measures such as early vaccination (16). Herein, we extend prior analyses for Germany (11) and for Italy (10) by exploring Standardized Mortality-Ratios (SMRs) for January–June 2020 by sex and age (<65 years and ≥65years) in 36 “regions” (in 16 German states and 20 Italian regions), comparing with mortality in previous years (12). In addition, we quantify the associated “burden of disease” for Germany and Italy (17, 18), i.e., we estimate associated changes of “summary measures of population health” (19) and focus on loss of life expectancy. Finally, we assessed to what extent partial mortality information from two German states (Hesse, Berlin) and from 19 Italian cities, regularly explored by the European monitoring of excess mortality for public health action network (EuroMOMO) (20, 21), represents each country as a whole. EuroMOMO monitors number of deaths per week from 26 participating European regions/countries (21).

Thus, our project aimed to answer three questions concerning the January–June 2020 first wave of the COVID-19 pandemic:

- What was the excess mortality (elevated SMR) for females and males of different age in Germany and Italy, and in their states and regions?
- What was the “burden of disease” for Germany and Italy?
- How does mortality (SMR) in Germany or Italy and its states or regions compare with partial state or city information?

## Methods

### Study Question (i)

For Germany, we analyzed all-cause mortality data by age (all ages, <65 years, ≥65 years) and sex (all sexes, female only, male only) published by the German Federal Statistical Institute (Destatis) (22) on 31 October 2020, specific for all 16 federal states from 1 January 2016 to 30 June 2020. For Italy, we analyzed data published by the Italian National Institute of Statistics (ISTAT) (23) on 10 August 2020, specific for all 20 regions for each day from 1 January 2016 to 30 June 2020. Death counts from Italy by sex were collapsed into age groups <65 years and ≥65 years. Mortality information was grouped by month and by week, thus facilitating investigation of temporal trends.

We calculated simplified SMRs with observed number of deaths in January–June 2020 (“observed”) and expected number of deaths (“expected”) as the average of those occurring in the same period in 2016–2019. SMRs were computed as the observed/expected ratio, with 95% confidence intervals (95%-CI) according to a Poisson distribution of death counts in 2020 (24, 25). We calculated arithmetic and geometric averages for 2016–2019 death counts, corresponding to a normal and lognormal distribution of death counts, respectively. We added variances from random errors within 2020 and between 2016 and 2019 on the log scale to derive extended 95%-CIs for SMRs, as recommended in random effects analysis (26), to account for the variation in death counts within 2020 and between the years 2016–2019. We note that our calculation of SMRs is simplified because the usual indirect standardization (24, 25) of the expected numbers by 5-year or 1-year age groups and by sex was not possible: the age distributions of the German populations were not available beyond 31 December 2019. In addition, the available categorization of death counts into two age groups was too coarse. This simplification may have led to biased estimates of SMRs if sizes and age-sex distributions change significantly between 2016 and 2020. However, it should not have affected substantially the comparison of SMRs across weeks in 2020 and by country, state, or region.

We used Poisson models with robust variance to estimate SMR changes across time and the influence of covariates/confounders (country, state/region, sex, age) on the SMR (24, 25, 27), also with over-dispersion parameters (negative binomial distributions) (28). SMR information was used in regression models taking both errors (“within” and “between”) simultaneously into account.

Different from EuroMOMO's regression fitting, which estimate a smoothed baseline mortality common to all years under observation to detect excess mortality (29), we contrasted mortality rates in 2020 with those from 2016 to 2019 using a ratio measure (SMR).

### Study Question (ii)

We estimated the change in mortality rate during January to June 2020 as (observed-expected)/Pop with Pop as the size of the population. The effect on life expectancy over such a short time-period is linked to the change in mortality rate [Appendix A in (30), Equation 19 and Appendix B in (31)]. The associated relative change in life expectancy was determined as 100^*^(expected-observed)/Pop (expressed as percentage, with a negative sign indicating loss of life expectancy). Absolute changes were calculated by multiplying the relative change with the remaining life expectancy of the population. We calculated extended 95%-CIs by transferring the extended 95%-CIs of SMRs to the observed numbers. To address potential overestimation of the expected deaths (e.g., due to pronounced influenza periods during the reference years) we determined the minimum of SMRs across weeks and biased the expected deaths downwards by multiplying with this minimum SMR so that the resulting adjusted SMR curve was always ≥1 (sensitivity analyses). The evaluations were performed for subpopulations in Germany and Italy separated by age (all ages, ≥65 years) and sex (both sexes combined, female, male) and in combination. Remaining life expectancy at mean age was used for the total populations, but at age 65 for the subpopulations of age ≥65 years (resulting in an overestimate of effects). Population data were extracted from publicly available data at the German Federal Statistical Institute (32–35) and the Italian National Institute of Statistics (36–39). We report findings as relative or absolute losses of life expectancy, i.e., as the negative of changes in life expectancy in % or days, respectively.

### Study Question (iii)

We contrasted January-June 2020 SMRs for Germany as a whole with SMRs for Berlin and Hesse, the only German states contributing to the EuroMOMO network (19, 20), and those for Italy as a whole with SMRs for 19 Italian cities, the sole Italian contributors to EuroMOMO (40).

All analyses were performed with Stata 14 (41). *P*-values <0.05 were defined as statistically significant.

## Results

### Study Question (i)

**Figure 1:**
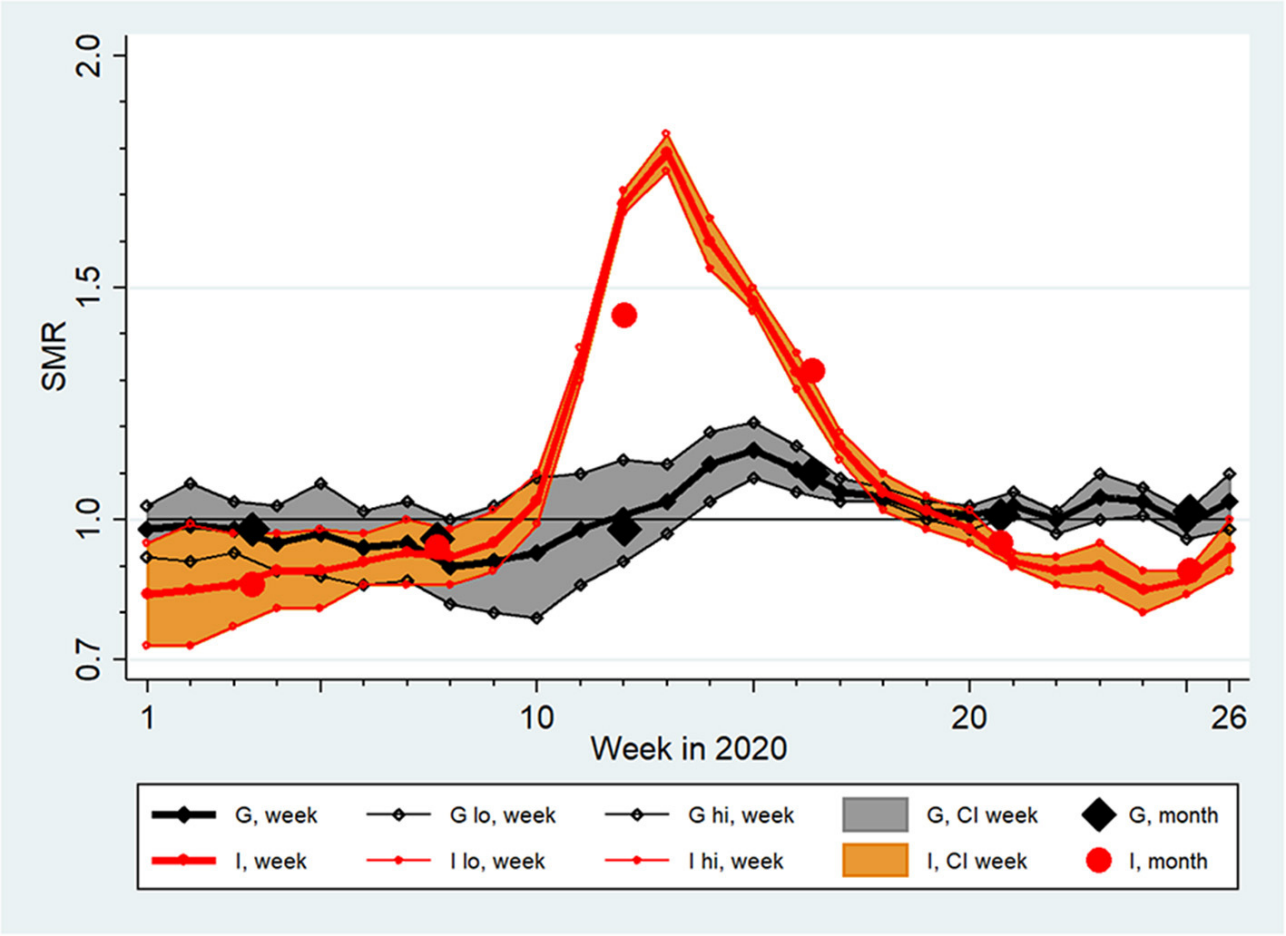
Standardized Mortality Ratios (SMR) with extended 95% confidence intervals (CI, lo, hi) by week and month in Germany (G) and Italy (I) from January (week 1) to June 2020 (week 26) assuming a lognormal distribution of country-specific baseline death counts between 2016 and 2019.

During the first 6 months of 2020, both Germany and Italy experienced significant increases in mortality (Germany: week 14–18, Italy: week 11–18; Figure 1). The SMR peaked in week 15 in Germany (1.15, 95%-CI: 1.09–1.21), and in week 13 in Italy (1.79, 95%-CI: 1.75–1.83). Overall, the SMR was 1.00 (95%-CI: 0.97–1.04) based on 484,762 observed deaths in Germany, and 1.06 (95%-CI: 1.03–1.10) based on 354,425 observed deaths in Italy.

**Figure 2:**
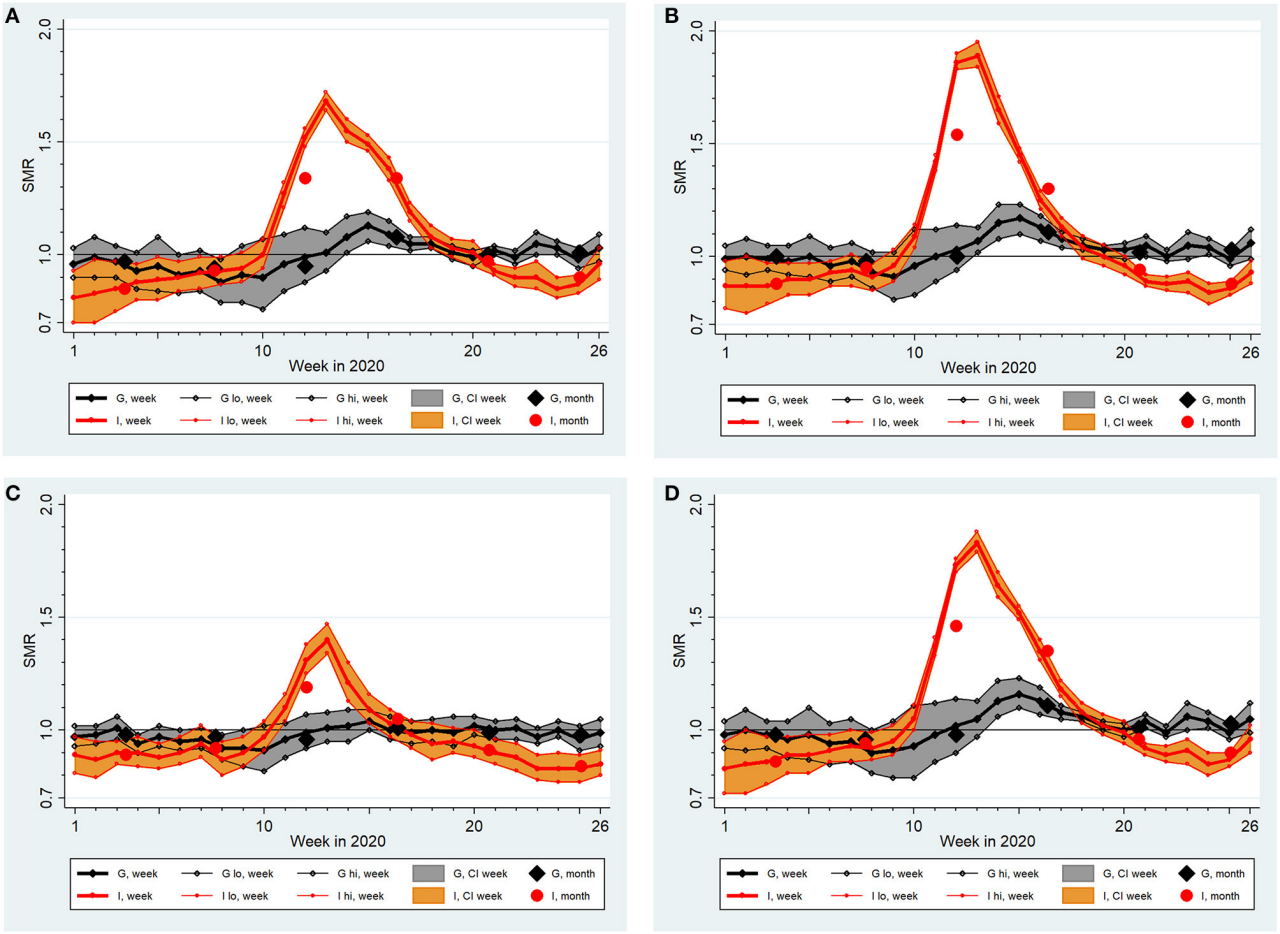
Standardized Mortality Ratios (SMR) with extended 95% confidence intervals (CI, lo, hi) by week and month in Germany (G) and Italy (I) from January (week 1) to June 2020 (week 26) assuming a lognormal distribution of country-specific baseline death counts between 2016 and 2019: **(A)** females, **(B)** males, **(C)** age at death <65 years, **(D)** age at death ≥65 years.

In both countries, SMR curves by sex and by age were qualitatively similar to the respective total populations displayed in Figure 1, but mortality excesses were clearly higher in males and in people of age ≥65 years (Figure 2). Overall, this elevation in males was not significant in Germany (SMR = 1.02, 95%-CI 0.99–1.06) but was significant in Italy (SMR = 1.08, 95%-CI: 1.04–1.11). The average SMR was also elevated significantly among Italian females (1.05, 95%-CI: 1.01–1.10). In Germany, there was no significant overall mortality excess in the total population aged ≥65 years (SMR = 1.01, 95%-CI: 0.97–1.05), nor after separation by sex. In contrast, an 8% increase in mortality for the total population aged ≥65 years was observed in Italy (SMR = 1.08, 95%-CI: 1.03–1.12), which was less pronounced among females (SMR = 1.06, 95%-CI: 1.01–1.11) than among males (SMR = 1.09, 95%-CI: 1.06–1-13).

In both countries the SMR for people younger than 65 years was neither elevated in the total populations nor in males or females. However, in Italy, the SMR increased up to 1.4 (95%-CI: 1.34–1.47) in week 13 (age <65 years, both sexes).

Overall and maximum weekly SMRs in January–June 2020 for each German state and Italian region are presented in Table 1. No German state but seven Italian regions showed significantly elevated overall SMRs; maximum SMRs showed a significant excess in 12 out of 16 German states and in 16 out of 20 Italian regions. Overall SMRs varied widely between states and regions, with a smaller span in Germany (Brandenburg: 1.04, Thuringia: 0.97) than in Italy (Lombardy: 1.48, Basilicata: 0.85).

**Table 1:** Standardized mortality ratios (SMR) with extended 95% confidence intervals (95%-CIs) for weeks 1–26 in 2020 in 16 states and 20 regions in Germany (G) and Italy (I), respectively, assuming a lognormal distribution of state/region-specific baseline death counts between 2016 and 2019.

**State/region**	**Country**	**SMR January–June**	**95%-CI**	**Highest SMR**	**95%-CI**
Lombardy	I	1.48	1.42–1.54	3.99	3.85–4.15
Trentino Alto Adige	I	1.17	1.13–1.22	2.37	2.14–2.62
Emilia Romagna	I	1.16	1.13–1.20	2.13	2.02–2.24
Aosta Valley	I	1.13	1.04–1.22	3.29	2.55–4.24
Liguria	I	1.12	1.07–1.17	2.10	1.95–2.26
Piedmont	I	1.11	1.07–1.15	2.08	1.93–2.24
Marche	I	1.05	1.01–1.10	1.85	1.71–2.01
Brandenburg	G	1.04	1.00–1.08	1.17	1.09–1.27
Bavaria	G	1.03	0.99–1.08	1.28	1.21–1.34
Veneto	I	1.02	0.99–1.04	1.43	1.35–1.52
Hamburg	G	1.02	0.98–1.06	1.25	1.08–1.44
Baden-Wuerttemberg	G	1.02	0.98–1.06	1.24	1.17–1.32
Bremen	G	1.01	0.95–1.08	1.31	1.09–1.58
Berlin	G	1.01	0.97–1.04	1.14	1.05–1.23
Hesse	G	1.00	0.97–1.04	1.15	1.05–1.25
North Rhine-Westphalia	G	1.00	0.96–1.03	1.13	1.07–1.20
Lower Saxony	G	1.00	0.97–1.04	1.12	1.04–1.20
Tuscany	I	0.99	0.95–1.02	1.28	1.19–1.38
Apulia	I	0.99	0.94–1.04	1.17	1.06–1.29
Mecklenburg Western-Pomerania	G	0.99	0.94–1.04	1.16	1.03–1.30
Saxony	G	0.99	0.94–1.04	1.11	1.02–1.20
Rhineland-Palatinate	G	0.99	0.95–1.03	1.07	1.00–1.15
Saarland	G	0.98	0.92–1.05	1.16	1.00–1.33
Saxony-Anhalt	G	0.98	0.93–1.03	1.15	1.05–1.26
Schleswig-Holstein	G	0.98	0.94–1.02	1.10	1.00–1.22
Friuli Venezia Giulia	I	0.97	0.94–1.01	1.31	1.17–1.46
Thuringia	G	0.97	0.92–1.02	1.10	0.99–1.21
Abruzzo	I	0.94	0.90–0.99	1.43	1.28–1.60
Umbria	I	0.94	0.89–0.99	1.22	1.05–1.42
Sardinia	I	0.94	0.90–0.98	1.18	1.05–1.32
Calabria	I	0.92	0.88–0.97	1.12	1.01–1.23
Molise	I	0.91	0.86–0.96	1.19	0.95–1.50
Campania	I	0.91	0.87–0.95	1.05	0.97–1.13
Latium	I	0.88	0.84–0.92	1.07	1.01–1.14
Sicily	I	0.88	0.84–0.91	1.04	0.96–1.13
Basilicata	I	0.85	0.80–0.90	1.01	0.82–1.23

Most German states and Italian regions showed an SMR curve similar to those presented in Figure 1 for Germany and Italy, but there were exceptions. In Mecklenburg-Western Pomerania, SMRs showed a constant increase leading to an excess mortality in week 24; in Basilicata, almost all SMRs indicated a non-significantly decreased mortality (maximum weekly SMR = 1.01) and there was no systematic SMR pattern across time.

**Figure 3:**
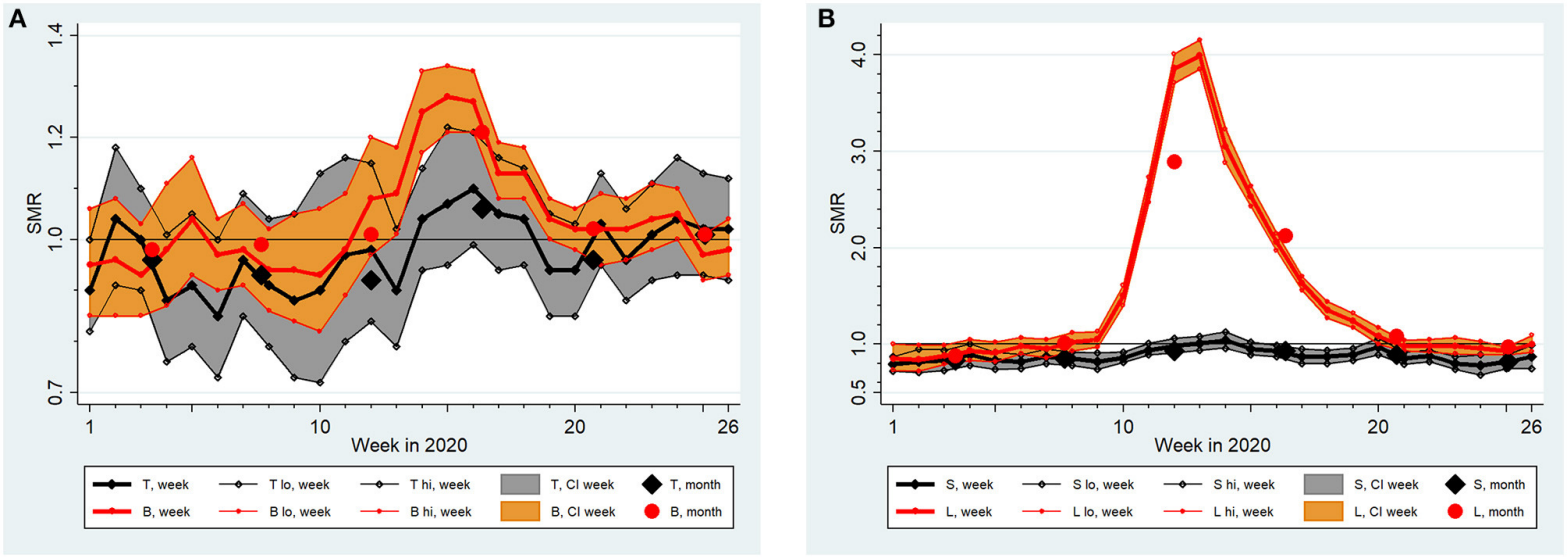
Standardized Mortality Ratios (SMR) with extended 95% confidence intervals (CI, lo, hi) by week and month in **(A)** Thuringia (T) and Bavaria (B) and **(B)** Sicily (S) and Lombardy (L) from January (week 1) to June 2020 (week 26) assuming a lognormal distribution of region-specific baseline death counts between 2016 and 2019.

Although SMR curves among many states and regions showed a similar pattern across time (increase, peak, decrease), the findings differ substantially, e.g., in maximum SMRs. This is exemplified in Figure 3 which displays the contrast between Thuringia and Bavaria in Germany and between Sicily and Lombardy in Italy. Excesses were obviously larger in Bavaria and Lombardy, in agreement with overall and peak SMRs differing between the respective states and regions (Table 1).

Table 2 summarizes findings from three negative binomial regression models: one joint analysis of both countries and two country-specific analyses. All models include “week” as a categorical variable with 26 levels to take account of the non-linear time development of SMRs. The modeling effectively repeated the curvatures shown in Figure 1. Age and sex were shown to be strong modifiers of SMRs in all models (see Figure 2 for a demonstration of the impact of age and sex). The “country x week” and “country x age” interaction terms used in the joint analysis demonstrate significantly higher SMRs in Italy due to modifications by these variables (not so “country x sex”). We did not include “country” as a covariate because this is well reflected by the categorical “region” variable (all 36 “regions” from both countries were included simultaneously). Irrespective of the adjustments performed, Table 2 demonstrates a highly significant heterogeneity of SMRs across regions in Italy and in Germany (*P* < 0.0005). To exemplify this heterogeneity, we report the factor with 95%-CI for Thuringia and Bavaria (cp. Figure 3A) estimated from the German model (Thuringia: 0.99, 95%-CI: 0.97–1.02; Bavaria: 1.06, 95%-CI: 1.03–1.08) and for Sicily and Lombardy (cp. Figure 3B) estimated from the Italian model (Sicily: 0.94, 95%-CI: 0.90–0.98; Lombardy: 1.43, 95%-CI: 1.32–1.54). The differences shown in Figure 3 are clearly significant because confidence intervals do not overlap. The overdispersion parameter estimate was >0 in all models and pronounced for Italy. Regardless, fitting Poisson models with equidispersion did not influence the results remarkably. Similar findings were returned after dropping the “robust variance” option, when the mortality data was aggregated into months, and when expected number of deaths were based on a normal distribution.

**Table 2:** Negative binomial regression analysis (with robust variance estimation) of standardized mortality ratios with extended 95% confidence intervals for weeks 1–26 in 2020 assuming a lognormal distribution of baseline death counts between 2016 and 2019.

	**Italy vs. Germany**		**Italy**		**Germany**	
	**Factor^a^ (indicators)**	***P*-value in %**	**Coefficient (indicators)**	***P*-value in %**	**Coefficient (indicators)**	***P*-value in %**
“Region”	(2–36)^b^	<0.05	(2–20)^b^	<0.05	(22–36)^b^	<0.05
Age^c^	1.032	<0.05	1.083	<0.05	1.037	<0.05
Country^d^ x Age	1.055	<0.05	-	-	-	-
Sex^e^	1.026	<0.05	1.020	3.9	1.030	<0.05
Week^f^	(2–26)	5.0	(2–26)	<0.05	(2–26)	<0.05
Country^d^ x Week^f^	(2–26)	<0.05	-	-	-	-
Alpha^g^	0.0112	-	0.0215	-	0.00040	-

a *Exponentiated regression coefficient or list of binary indictor variables (in parentheses)*.

b *Italy/Germany: 36 “regions” (reference: 1), Italy: 1-20 (ref: 1), Germany: 21-36 (ref: 21)*.

c *≥65 years vs. <65 years (ref)*.

d *Italy vs. Germany (ref), x: interaction term*.

e *Male vs. female (ref)*.

f *Weeks 1–26 in 2020 (ref: week 1)*.

g *Overdispersion parameter estimate*.

### Study Question (ii)

We report burden-of-disease findings as estimated losses of life expectancy during the first half of 2020 in Table 3, based on weekly mortality data and assuming a lognormal distribution of death counts between 2016 and 2019. The relative loss of life expectancy in Germany was 0.0025% for the total population and 0.012% for the male subpopulation. These estimates correspond to an absolute loss of 0.34 and 1.66 days, respectively. In Italy, the loss was significant and more pronounced: 0.035% for the total population and 0.042% in males, corresponding to 5.3 and 6.3 days, respectively. Restricting the analyses to deaths at age ≥65 years gives estimated relative losses in Germany of 0.020% (95%-CI: −0.07, 0.11%) for the total population and 0.074% (95%-CI: −0.02, 0.19%) in males; in Italy, estimated relative losses were 0.16% (95%-CI: 0.05, 0.25%) for the total population and 0.21% (95%-CI: 0.14, 0.30%) in males. Applying upper values of remaining life expectancy by using the values at the minimum age of 65 years, upper limits of corresponding absolute losses were 1.4 days for the total population (males: 4.8 days) in Germany and 13.7 days for the total population (males: 16.7 days) in Italy. Burden estimates based on a normal distribution of death counts between 2016 and 2019 or analyzing monthly data did not differ remarkably.

**Table 3:** Estimated loss of life expectancy by country and sex from January to June 2020 using mortality from January to June 2016–2019 as reference (lognormal distribution, weekly mortality data) with 95% confidence intervals (95%-CI).

	**r^a^LLE^b^ (95%-CI)**	**a^c^LLE (95%-CI)**
**Germany**		
Total	0.00 (−0.02, 0.03)	0.34 (−2.07, 3.55)
adj^d^	0.06 (0.04, 0.08)	8.34 (5.93, 11.55)
Female	−0.01 (−0.03, 0.02)	−1.01 (−4.29, 2.27)
adj	0.06 (0.04, 0.09)	8.86 (5.58, 12.14)
Male	0.01 (0.00, 0.04)	1.66 (−0.67, 4.78)
adj	0.06 (0.05, 0.09)	8.66 (6.33, 11.78)
**Italy**		
Total	0.04 (0.02, 0.06)	5.32 (2.80, 8.68)
adj	0.12 (0.11, 0.15)	18.72 (16.20, 22.08)
Female	0.03 (0.01, 0.06)	4.37 (0.91, 8.70)
adj	0.14 (0.11, 0.16)	20.81 (17.35, 25.14)
Male	0.04 (0.02, 0.06)	6.33 (3.09, 8.76)
adj	0.13 (0.11, 0.15)	19.31 (16.07, 21.74)

a *r, relative in %*.

b *LLE, loss of life expectancy*.

c *a, absolute in days*.

d *adj (adjusted), upwardly biased estimates assuming lowest weekly SMR <1 as reference*.

### Study Question (iii)

For the German component, we fitted models restricted to Germany that included an indicator variable for states Hesse and Berlin combined, and an interaction term of this indicator with the categorical week variable. The overall SMR for Hesse and Berlin was about 1–2% higher than the SMR for Germany without Hesse and Berlin (not significant); the increase in the April SMR was lower in the two states (~2%). The SMR curve in Hesse and Berlin was thus structurally different from that of the other federal states in Germany. This different course of SMRs in Hesse and Berlin vs. Germany without these two states was evident in the weekly and monthly data and was clearly significant in the weekly data (interaction: *P* = 0.016). We note that Hesse and Berlin accounted for only 10.8% of all observed deaths in Germany during January to June 2020.

For the Italian component, we extracted observed and expected numbers of deaths for the 19 Italian cities (i.e., EuroMOMO's Italian data base) from two reports (42, 43). Observed and expected numbers were available (almost completely with few days missing) for the period February to June 2020. We compared these data with mortality data for Italy as a whole during the same period. The 19 cities accounted for ~11% of the observed deaths in Italy. The SMR was 14% higher than in Italy as a whole. This overestimate of the SMR was statistically significant—a Poisson regression model estimated the 95%-CI of the excess in the 19 cities as 13 to 15%, *P* < 0.0005. Other modeling approaches (Poisson model with robust variance estimation, negative binomial regression with or without robust variance estimation) confirmed this finding. We note that expected numbers of deaths were based on five reference years in the two reports (42, 43), whereas numbers for whole Italy were based on four reference years.

## Discussion

Our analysis of mortality during the SARS-CoV-2/COVID-19 pandemic focused on SMRs in Germany and Italy between January and June of 2020 and included population subcategorization by state/region, sex, and age. Thus, it allows comparing overall mortality between and within the countries, over time, and in different subpopulations.

Regarding study question (i), that Italy was hit earlier and much harder than Germany in the first half of 2020 was expected and corroborated by our systematic analyses (Table 2; Figures 1–3). Important insights were provided by higher spatial resolution through the analyses of 16 German states and 20 Italian regions. Stang et al. (11) argued that country-specific analyses need to complement pooled analyses across Europe (20). Our work shows that an aggregate look at a country can mask mortality changes in smaller areas of observation, which convey essential public health information. While the selected contrast “region” pairs in Figure 3 share qualitatively similar courses of SMR increases, they are very different quantitatively. Such information is key to understand the dynamics and conceivable risk mosaic of SARS-CoV-2/COVID-19-associated mortality over space and time. This is important in countries like Germany where federal states can enact policy different to recommendations made at the country level or like Italy where a regional tiered-approach as a mitigation strategy was enacted. Moreover, in both countries, increased SMRs for males and individuals above 65 years were detected. These differences show that separate statistics for different subgroups are needed to assess actual risks and to inform targeted prevention measures.

Regarding study question (ii), publications frequently focus on “excess deaths” (i.e., the difference between observed and expected deaths) to describe the potential effect of COVID-19 on mortality of a population (“burden of disease”) (20, 44, 45). This statistic suffers from three drawbacks. First, it depends on the size of the population studied, so comparisons across regions or countries are potentially biased. Second, if baseline death counts vary with season (and they do), excess deaths vary with this background even if the effect is the same in each individual. Third, this statistic conveys the wrong intuition that numbers of excess deaths were numbers of deaths due to COVID-19. Even if biases can be excluded, both figures can be far apart (18). In contrast, mortality rates are the “force of mortality” (25) and the ratio of mortality rates measures the change of this force due to exposure. Whereas deaths due to COVID-19 cannot be estimated from excess deaths without making unrealistic assumptions, changes in mortality rates can be transferred reliably into changes in life expectancy as an appropriate burden-of-disease measure (18).

Our estimates of loss of life expectancy during the first half of 2020 differ remarkably between the German and Italian populations (Table 3). This is in line with our findings about SMRs presented in Figure 1 and regression modeling results in Table 2. In addition, males suffered from larger losses than females, corresponding to differences in SMRs by sex as shown in Figure 2 and to significantly elevated regression coefficients among males (Table 2). Yet, our estimates of lost life expectancy are rather small. Among German and Italian males and females, the Italian males showed highest SMRs but the absolute loss was clearly smaller than 10 days in this group, i.e., definitely <0.1% of their remaining life expectancy (Table 3). This is small in comparison to loss of life expectancy due to lifestyle risk factors in German males, e.g., smoking more than 10 cigarettes/day (about 9 years of life lost), consuming more than 4 alcoholic drinks/day (about 3 years), or obesity, i.e., BMI>30 kg/m^2^ (about 3 years) (46). Of note, the exposure to SARS-COV-2 is over a short time-period (so far) while lifestyle factors are long-term exposures. In addition, the performed mortality comparison between 2020 and 2016–2019 might divert from the counterfactual difference between mortality in 2020 and the mortality we would have observed if COVID-19 had been absent. For instance, mortality in the reference years may have been driven upward by severe influenza periods not occurring in 2020. To address for this potential overestimate of expected deaths, we performed sensitivity analyses involving intentionally upward biased SMRs. The adjustment increased the estimates of absolute losses of life expectancy to 8.3 days for Germany and to 18.7 days for Italy, with an upper 95%-CI limit for Italian males of 25 days (Table 3). We conclude that the average burden of disease was probably < ~1–2 weeks of lost life expectancy in Germany and < ~2–3 weeks in Italy due to COVID-19 during the first 6 months of 2020.

Restricting analyses to deaths at age ≥65 years led to the relative losses of life expectancy increasing substantially by a factor of 4–8 in comparison to the average burden of the whole population and in males (see section Results). Adjusting the estimates in a biasing sensitivity analysis increased the estimated relative losses by a further factor 4–12 in Germany and 2–3 in Italy. We applied upper values of remaining life expectancy by using the values at the minimum age of 65 years to derive upper limits for the absolute losses. The combined procedure (upward adjustment by sensitivity approach and by maximum remaining life expectancy) resulted in upper bounds of absolute losses among the older populations (age ≥65 years) and similar in both sexes of 18 days (95%-CI: 11–24) in Germany and 45 days (95%-CI: 35–52) in Italy. Taken together, the average loss of life expectancy among the older population (age ≥65 years) was < ~3 weeks in Germany and < ~6 weeks in Italy due to COVID-19 during the first 6 months of 2020. Results from sensitivity analyses based on a normal distribution of death counts within 2016–2019 were similar albeit less pronounced when monthly data were used because minimum SMRs across weeks were smaller than across months.

“Burden of disease” must be interpreted in context. First, the estimates of loss of life expectancy refer to the total populations of Germany and Italy; specific victims will have experienced wide ranges of individual loss of life expectancy. Moreover, this burden due to COVID-19 is carried completely by the infected subpopulation, which comprised probably <10% of the total population in Germany and Italy until end of June 2020 (47–49). Assuming that the calculated relative loss in life expectancy among the populations would stem from the fraction of the infected only, the estimated relative loss among the infected would be much larger (it is inversely proportional to that fraction). Second, these observations were made with massive counter-measures taken. It would be remiss to speculate how many deaths and how much loss of life expectancy could have resulted from hospital overcrowding in Germany or Italy that was avoided or mitigated by radical actions. Third, our study merely assessed deaths in the first half of 2020. Possible medium- and long-term effects on life expectancy of those with SARS-CoV-2 infections who survived from January to June of 2020 are not appreciated in our calculations of “burden of disease.”

Regarding study question (iii), we note that Hesse and Berlin which serve as the database for Germany in EuroMOMO (20) accounted for ~11% of all deaths that occurred in Germany between January and June 2020. The SMR curve for Hesse and Berlin was structurally different from that of the other states in Germany. This different course of SMR in Hesse and Berlin vs. Germany without Hesse/Berlin was clearly evident in the weekly data. Supported by the general finding of a pronounced variation of SMRs across “regions” (Table 2), confining surveillance to two states may lead to bias. Data from “19 cities” which provide mortality data to the EuroMOMO “country-pool” on a weekly basis (20) accounted for ~11% of the observed deaths in Italy, suggesting that the SMR in the 19 cities is about 13% higher than in Italy as a whole. This difference in SMR is statistically significant. Summarizing our empirical results, data feeds from Hesse and Berlin and from “19 cities” neither represent the two countries' mortality as a whole nor in their states and regions from January through June 2020, at least not in this instance. In effect, EuroMOMO's approach to estimate what happens on a country level (and, after pooling, on a European level) needs discussion. Empirically, EuroMOMO lacks data representing the country level of Germany and Italy. Moreover, even if they had whole country level information, these data are not reliable to understand what has happened—and are not reliable to decide what should be invoked as countermeasures—at regional levels within Germany and Italy in 2020.

A general consideration for mortality analyses is the change in age structures over time. zur Nieden et al. (45) discussed the problem of missing age adjustment and explained the trend toward an older population in Germany from 2016 to 2019. Our evaluation of the change in age structure between 2016 and 2019 (35) showed an increase of the German population from 82,521,653 as of 31 December 2016 to 83,166,711 as of 31 December 2019 (+0.78%). The relative increase was larger in males (+0.84%) than in females (+0.73%), and in older (+3.32%) than in younger people (+0.10%), and particularly in males aged ≥65 years (+4.06%) compared to females aged ≥65 years (+2.74%). Our analysis of Italian population data (38) showed a decreasing population from 60,589,445 as of 31 December 2016 to 60,244,639 as of 31 December 2019 (−0.57%). This decrease was less pronounced in males (−0.36%) than in females (−0.77%). Of note, the number of people aged ≥65 years increased by +3.10%. This was more pronounced in males (+3.99%) than in females (+2.41%). In our view, the estimated changes in age-sex structures of the populations cannot explain observed differences in SMRs during 2020 within either country, or between Germany and Italy. Empirical evidence, supported by this study, suggests that COVID-19-associated mortality is higher in males than in females, and higher in older than younger people both in Germany and in Italy (10). Moreover, numbers of deaths are mainly driven by older people. Thus, SMRs as calculated in our study are potentially overestimated in Germany and Italy (despite a decreasing population segment) because we cannot adjust for age and sex appropriately. However, and importantly, they do not affect comparisons of SMRs substantially. We note that the amount of bias may vary within countries because changes were heterogeneous across states in Germany and regions in Italy (*p* < 0.05 always).

A recent report (50) explored mortality in Italy from February-May 2020, with an interrupted time-series analysis, modeling daily figures with smooth spline functions, and with a finer spatial resolution by aggregating data at the province level, and adjusting by age. The authors raised the point that deriving expected events from past years would not account for time trends in mortality and changes in the average temperature and the seasonal influenza epidemic. As previously discussed, we do not see a remarkable effect of missing age adjustment. As it concerns the effect of the weather conditions and the influenza pandemic, mentioned in (50), a recent geographic correlation study (51) explored their effect on COVID-19 cumulative incidence, mortality, and case-fatality rate. The authors did not observe an association between average March 2020 regional temperature and COVID-19 cumulative incidence, mortality, and case fatality rate. Instead, influenza vaccination rate in the elderly was inversely related to the three COVID-19 outcomes, and particularly mortality and case-fatality rate.

As for the analytical strategy, regression analysis “encompasses a vast array of techniques designed to overcome the numerical limitations of simpler methods. This advantage is purchased at a cost of stronger assumptions (25).” The regression model used in (50) constrains the excess mortality to start from null at the beginning of February whereas this excess mortality is shown to be negative (see Figures 1, 2A–D of this paper), and this model lead to underestimating the mortality ratio in the following weeks [see upper panel in Figure 3 of (50)].

The question “What effects did counter-measures have on mortality?” was beyond the scope of our study for several reasons. Compliance with counter-measures is uncertain. Reliable and comparable data on key contributors to the necessary mix of counter-measures such as systematic preparedness, including testing or contact tracing, are not yet available for Germany and Italy. Future work should explore the role of specific counter-measures, some of which might have had potential to harm rather than help (52).

We note further that it is not possible to adjust estimates of the effect of counter-measures for the potential confounding by seasonality of coronaviruses appropriately as demonstrated by Figure 1 of (53) and Figure 5 in (54). Prevalence of coronaviruses decreases between February and April, almost collinear to the reduction of observed mortality excesses when taking a latency of 3–4 weeks between infection and death from COVID-19 into account.

Overall, our work showed differential excess mortality for females and males of different age in Germany and Italy, with substantial variation by individual states and regions. Therefore, our work serves as proof-of-principle that use of publicly available mortality information by independent researchers can result in informative analyses for policymakers. Moreover, our findings challenge the use of partial mortality information for the two countries in EuroMOMO's international mortality database. We conclude that European and national institutions should explore and publish routinely collected mortality data with appropriate temporal and spatial resolution (55) to understand past, and to prepare for future, challenges (17).

## Data Availability Statement

Publicly available datasets were analyzed in this study. This data can be found at Destatis. Sterbefälle–Fallzahlen nach Tagen, Wochen, Monaten, Altersgruppen und Bundesländern für Deutschland 2016–2020, 2020, 12.11.2020. Available from: https://www.destatis.de/DE/Themen/Gesellschaft-Umwelt/Bevoelkerung/Sterbefaelle-Lebenserwartung/Tabellen/sonderauswertung-sterbefaelle.html. ISTAT. Istat during the Covid 19 emergency, 2020; Available from: https://www.istat.it/.

## Author Contributions

PM and TE conceived the study. PM, TE, BT, JG, and PC organized the project. PM, TE, and PC designed the study. PM, BT, JG, and PC acquired and organized data for the analyses. PM conducted statistical analyses. PM, TE, JG, PL, BT, and PC worked on the tables and figures. PM, TE, BT, JG, PL, and PC interpreted results. TE and PM prepared the original draft. PL and PC contributed to critical revision of the manuscript. All authors reviewed, commented on the manuscript draft, approved the material submission, and are accountable for the work. PM is guarantor, takes full responsibility for the accuracy of the data analyses, and attests that all listed authors meet authorship criteria.

## Conflict of Interest

The authors declare that the research was conducted in the absence of any commercial or financial relationships that could be construed as a potential conflict of interest.
